# Amputation-free survival in 17,353 people at high risk for foot ulceration in diabetes: a national observational study

**DOI:** 10.1007/s00125-018-4723-y

**Published:** 2018-08-31

**Authors:** Thenmalar Vadiveloo, William Jeffcoate, Peter T. Donnan, Helen C. Colhoun, Stuart McGurnaghan, Sarah Wild, Rory McCrimmon, Graham P. Leese, on behalf of the Scottish Diabetes Research Network Epidemiology Group

**Affiliations:** 1 Division of Population Health Sciences, Medical Research Institute, University of Dundee, The Mackenzie Building, Kirsty Semple Way, Dundee, DD2 4BF UK; 2 Nottingham University Hospitals Trust, Nottingham, UK; 3 Institute of Genetics and Molecular Medicine, University of Edinburgh, Edinburgh, UK; 4 Usher Institute for Public Health Sciences and Informatics, University of Edinburgh, Edinburgh, UK; 5 Division of Molecular and Clinical Medicine, University of Dundee, Dundee, UK

**Keywords:** Amputation, Diabetes, Foot, Mortality, Ulcer

## Abstract

**Aims/hypothesis:**

Our aim was to investigate amputation-free survival in people at high risk for foot ulceration in diabetes (‘high-risk foot’), and to compare different subcategories of high-risk foot.

**Methods:**

Overall, 17,353 people with diabetes and high-risk foot from January 2008 to December 2011 were identified from the Scotland-wide diabetes register (Scottish Care Information-Diabetes: *N* = 247,278). Participants were followed-up for up to 2 years from baseline and were categorised into three groups: (1) those with no previous ulcer, (2) those with an active ulcer or (3) those with a healed previous ulcer. Participants with prior minor or major amputation were excluded. Accelerated failure time models were used to compare amputation-free survival up to 2 years between the three exposure groups.

**Results:**

The 2 year amputation-free survival rate in all people with diabetes with high-risk foot was 84.5%. In this study group, 270 people (10.0%) had an amputation and 2424 (90.0%) died during the 2 year follow-up period. People who had active and healed previous ulcers at baseline had significantly lower 2 year amputation-free survival compared with those who had no previous ulcer (both *p* < 0.0001). The percentage of people who died within 2 years for those with healed ulcer, active ulcer or no baseline ulcer was 22.8%, 16% and 12.1%, respectively.

**Conclusions/interpretation:**

In people judged to be at high risk of foot ulceration, the risk of death was up to nine times the risk of amputation. Death rates were higher for people with diabetes who had healed ulcers than for those with active ulcers. However, people with active ulcers had the highest risk of amputation.

**Electronic supplementary material:**

The online version of this article (10.1007/s00125-018-4723-y) contains peer-reviewed but unedited supplementary material, which is available to authorised users.

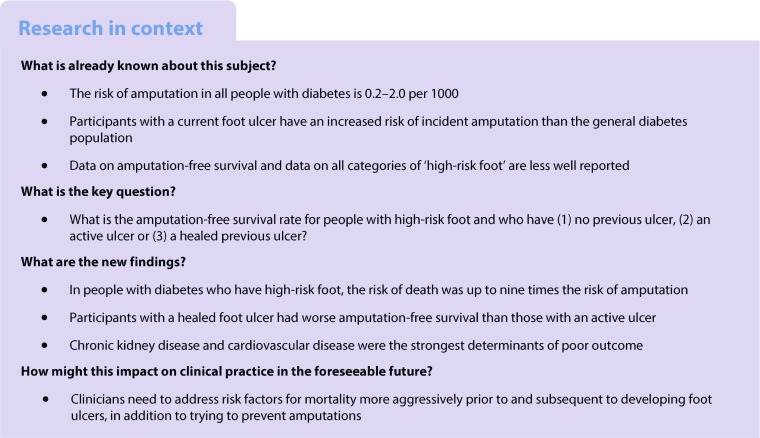



## Introduction

Diabetic foot ulcers and amputations are devastating and much feared complications of diabetes. Between 15% and 34% of people with diabetes develop a foot ulcer during their lifetime, with more than half acquiring infections [1] that may result in lower extremity amputations causing disability, extensive periods of hospitalisation, and premature mortality [2, 3]. The incidence of major amputation ranges from 0·2 to 2·0 per 1000 people in those with diabetes [4, 5]. Major or minor amputation also increases the risk of additional subsequent amputations [6]. Foot ulcers are the costliest microvascular complication of diabetes [7].

Amputations in people with diabetes have a significant impact on ambulation, body care, movement and mobility, resulting in an inability to perform daily tasks and often a loss of employment [6] impacting on the wider family. Clinical epidemiology studies suggest that foot ulcers precede around 85% of non-traumatic lower extremity amputations in individuals with diabetes [8] and hence ulcer prevention is important. Previous studies have reported that apart from severity of ulcer, age [9], low socioeconomic status, smoking [10, 11], sex [12], renal impairment [13], ischaemic heart disease, diabetic neuropathy [14], glucose levels [15] and peripheral arterial disease [16] are some of the important factors associated with the risk of amputation. Identifying a person’s risk of foot ulceration helps in directing scarce resources to those most at need. Assessment of individual risk factors can be used to determine an overall risk score for a person with diabetes; several systems have been developed and are used in routine clinical practice [17–21]. Most studies have identified a history of a previous ulcer as the strongest predictor of future ulcers [17–20, 22], although a global risk score is a more sensitive way of predicting foot ulceration than using any individual risk score [23].

Clinical tools are used to predict which individuals with active ulcers are at greatest risk of amputation, including the Wagner, University of Texas, SINBAD and other scores [24]. For people with diabetes who have an active ulcer, the final healing rates are 65–75% for those attending a hospital clinic, while around 15–20% of all people with an ulcer undergo amputation [18, 25–28], depending on duration of follow-up. Although outcomes have been reported for all people with diabetes, ulcers and even those with individual risk factors, there are no reported outcomes for people identified as having ‘high-risk foot’. Additionally, it is recognised that people with diabetes who have active foot ulcers are as likely to die as to undergo amputation [18, 25, 26], making amputation-free survival a more useful clinical outcome than amputation alone [26]. The aim of this study was to investigate the outcome of all people with diabetes who had ‘high-risk foot’, using amputation-free survival rate as the primary outcome. The factors associated with the risk of amputation in this group of individuals were also investigated in a secondary analysis.

## Methods

### Data sources

The Scottish Care Information-Diabetes (SCI-Diabetes) is a national population-based database which was established in 2000. This database is populated by daily downloads from primary and secondary care databases and contains demographic and clinical information covering over 99% of people with a diagnosis of diabetes in Scotland. At the time of the study there were 247,278 people with diabetes registered. The SCI-Diabetes database was linked anonymously to the national hospital admissions data (the Scottish Morbidity Record SMR01) and mortality data, which were provided by the Information Services Division (ISD) of National Health Service (NHS) and National Records of Scotland. Approval for generation and analysis of the linked dataset was obtained from the Caldicott guardians of all Health Boards in Scotland, the Privacy Advisory Committee of the Information Services Division of NHS National Services Scotland (ISD) and the national Multi-centre Research Ethics Committee.

### Study population

From the SCI-Diabetes database (>95% complete for foot recordings), people with diabetes who had been scored as ‘high risk’ for foot ulceration based on SIGN Guideline 116 criteria [17] between January 2008 and December 2011 were included. Study entry was the first record of ‘high risk’. In Scotland, individuals are classified as ‘high risk’ if they meet the following criteria: (1) they have had previous ulceration or amputation; (2) they have both absent pulses and inability to feel a 10 g monofilament or (3) they have one of the conditions in (1) or (2) together with callus or deformity. Greater detail is published elsewhere [17, 18]. Hereafter in this paper, such individuals are described as having ‘high-risk foot’. To limit confounding between the exposure groups, only incident amputations (either minor or major) were included. Thus, people with diabetes who had any (minor or major) amputation prior to the ‘high risk’ score were excluded from the study (*n* = 6654 [1.6%]). People with diabetes who had a high-risk foot on annual screening were then categorised into one of three exposure groups according to baseline data: (1) people with diabetes with no history of a foot ulcer; (2) those with healed ulcers and (3) those with active ulcers. The ‘no ulcer’ group had no history of any previous foot ulcer but had sufficient risk factors to classify them as ‘high risk’ [17]. The ‘healed ulcer’ group had a previous foot ulcer that was completely healed at baseline, while the ‘active ulcer’ group had a foot ulcer at baseline.

### Study variables

Information recorded at diagnosis of diabetes, such as date of birth, sex and date of diagnosis, were used in the study. HbA_1c_ measurement taken closest to baseline date, drug treatment for diabetes (insulin/tablets and glucagon-like peptide-1 [GLP-1] agonists) and eGFR measurement taken closest to baseline date were also included in the study. We did not have reliable data on smoking, diet or physical activity and the vast majority of participants (>95%) were from European descent, making it difficult to examine the impact of ethnic variation. Record linkage allowed identification of people with diabetes who had a history of cardiovascular diseases, using the International Classification of Diseases, 10th Edition (ICD-10) codes (http://apps.who.int/classifications/icd10/browse/2016/en) from hospital records. People with diabetes who had at least one amputation (either minor or major) or who died during the 2 years of follow-up were also identified.

### Statistical methods

Characteristics of people with diabetes and who had a high-risk of foot ulceration were described. The primary outcome for people with diabetes who had a high-risk of foot ulceration was amputation or death. Kaplan–Meier survival curves were plotted showing survival probabilities over the whole follow-up period in the three groups of people with diabetes. Cox proportional hazards model was used to compare hazards between the three exposure groups, adjusting for all the covariates. The assumption of proportional hazards was assessed by plotting log negative log plots for the baseline covariates and fitting time interactions. The assumption of proportional hazards for the exposure variable was violated (see electronic supplementary material [ESM] Table 1, ESM Figs 1 and 2). Therefore, the survival analysis was conducted using parametric regression models (accelerated failure time) to compare amputation-free survival up to 2 years between the three exposure groups. The endpoint included any major amputation (e.g. above ankle amputation) or minor amputation (e.g. below the ankle). The starting point was taken as the date of first record of high-risk of ulceration feet within the study period. The endpoint for each person with diabetes was whichever one of the following events came first: end of follow-up (2 years from baseline), death or amputation.

Accelerated failure time (AFT) models were used to examine demographic and clinical factors associated with 2 year survival without amputation for the three groups using *χ*^2^. A *p* value <0.05 was considered significant. AFT models with different distributions were fitted, including the generalised γ, log-logistic, log-normal, Weibull and exponential distributions. The Akaike’s information criterion (AIC) was used to select the optimal model (ESM Table 2). The unadjusted and adjusted estimates of coefficients and 95% CIs are reported. The models were adjusted for age, sex, duration of diabetes, HbA_1c_, eGFR, history of cardiovascular diseases and treatment for diabetes. A secondary analysis was done using amputation alone as event. Cox proportional hazards model was used to model the data as the assumption of proportional hazards was not violated. All analyses were conducted in SAS version 9.3 (SAS Institute, Cary, NC, USA) using PROC LIFEREG for AFT models and PROC LIFETEST to plot the Kaplan–Meier estimates.

## Results

### Baseline characteristics

Baseline characteristics of participants with high-risk foot (and no ulcer, active ulcer or healed ulcer) are shown in Table 1.

**Table 1:** Baseline characteristics of people with high-risk foot screening assessment and main outcomes

Covariate	All	No ulcer	Active ulcer	Healed ulcer	*p* value^a^
Participants, *n*	17,353	13,206	1731	2416	
Age, years	70.1 (12.2)	70.36 (12.1)	67.74 (12.5)	70.14 (12.2)	<0.001
HbA_1c_, mmol/mol	61.1 (19.5)	60.28 (19.2)	66.5 (20.9)	62.0 (19.4)	<0.001
HbA_1c_, %	7.7	7.6	8.2	7.8	
Duration of diabetes, months	115 (58–192)	103 (50–178)	156 (92–237)	152 (92–234)	<0.001
Treatment, %					
Lifestyle	41.9	42.7	39.7	38.9	<0.001
Tablet/GLP-1 agonist	29.8	31.6	21.7	26.3	
Insulin	28.3	25.7	38.6	34.8	
History of CVD, %	39.7	40.5	36.2	37.6	<0.001
Sex, % male	56.1	55.8	60.0	54.7	0.001
eGFR, %					
<30 ml min^−1^ [1.73 m]^−2^	5.2	4.7	7.3	6.6	<0.001
30–60 ml min^−1^ [1.73 m]^−2^	59.2	60.4	51.0	58.4	
>60 ml min^−1^ [1.73 m]^−2^	35.6	34.8	41.7	35.0	

Data are shown as mean (SD) or median (interquartile range), unless stated otherwise

^a^ANOVA for age, Kruskal–Wallis for duration of diabetes, *χ*^2^ test for the other categorical variables. Differences between the three groups (no ulcer, active ulcer and healed ulcer) were classified as significant if *p* < 0.05

CVD, cardiovascular disease

There were a total of 17,459 people with diabetes across Scotland who were identified with high-risk of feet ulceration from January 2008 to December 2011. Of these people, 106 had a record of both an active foot ulcer at baseline and healed ulcer prior to baseline. These people were excluded from the study as they did not fit into a single baseline study group. A total of 17,353 people with diabetes and high-risk foot were included in the study and, of these people, 13,206 (76.1%) had neither ulcers nor history of previous ulceration at baseline, 1731 (10.0%) people had active ulcers at baseline and 2416 (13.9%) people had healed previous ulcers at baseline. These people were followed-up for up to 2 years from baseline. The mean duration of follow-up was 22 months (SD 5.1).

Overall, 56.1% of the 17,353 people in the study sample were male and the mean age was 70.1 years (SD 12.2). There were significant differences between the three groups at baseline in terms of age, HbA_1c_, duration of diabetes, type of diabetes treatment, proportion with history of cardiovascular disease, sex distribution and eGFR. At baseline, people with active foot ulcers were significantly younger (*p* < 0.001), had higher HbA_1c_ (*p* < 0.001) and a greater proportion were men (*p* = 0.001), when compared with people with previously healed ulcers.

A total of 2694 (15.5%) people with diabetes had an amputation or died during the 2 year follow-up period. Of these, 270 (10.0%) had an amputation and 2424 (90.0%) died during the follow-up period. Although the percentage of events was highest in people with healed ulcer, the percentage of people who had an amputation was highest in the active ulcer group. Overall, the percentage of people who died was 14.0% in all high-risk individuals, 22.8% in those with previously healed ulcers, 16% in those with an active ulcer and 12.1% in those with no previous ulcer. The corresponding percentages of people who had an amputation were 1.6%, 1.0%, 2.4% and 1.5%.

### AFT model for those identified as having high-risk foot

**Fig. 1:**
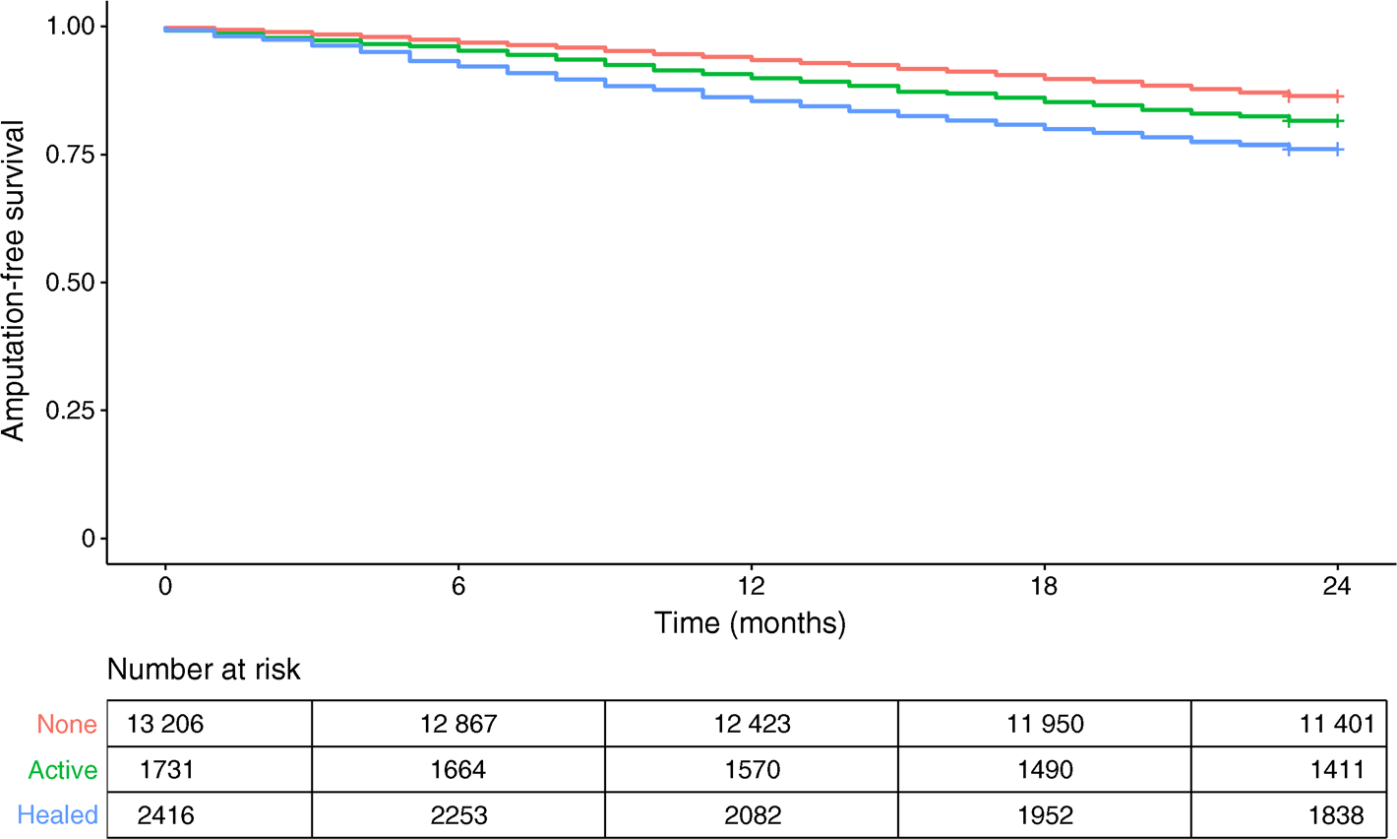
Kaplan–Meier survival curve showing amputation-free survival in people identified as having high-risk of foot ulceration. The healed ulcer group had the lowest amputation-free survival rate compared with the group with no previous or current ulcer and the active ulcer group. Red line, no ulcer; green line, active ulcer; blue line, healed ulcer

The crude 2 year amputation-free survival rate in all people with diabetes who had high-risk foot was 84.5%. Amputation-free survival was 85.0% in those with no previous or current ulcer, 81.6% in the active ulcer group and 76.1% in the healed ulcer group. The log-logistic regression model had the lowest AIC. The unadjusted and adjusted variable estimates for the final AFT model with log-logistic distribution are reported in Tables 2 and 3. We found that there was a shorter amputation-free survival time (i.e. worse outcome) for people who had active ulcers (*p* < 0.0001) or healed ulcers (*p* < 0.0001) at baseline compared with those who never had an ulcer, after adjusting for all variables. We also found that people with diabetes who were older, were male sex, had longer duration of diabetes, were treated with tablets or insulin, had higher HbA_1c_ level, had a history of cardiovascular disease and an eGFR lower than 30 ml min^−1^ [1.73 m]^−2^ had a significantly shorter amputation-free survival time. Figure 1 shows Kaplan–Meier curves for the outcome stratified by these exposure groups.

**Table 2:** Unadjusted and adjusted estimates for amputation-free survival in people identified as having high-risk of foot ulceration

Ulcer status	Population, n	Events, *n* (%)	Unadjusted time ratio (95% CI)	Adjusted time ratio^a^ (95% CI)
Active ulcer	1731	319 (18.4)	0.74 (0.66, 0.82)*	0.71 (0.63, 0.79)*
Healed ulcer	2416	577 (23.9)	0.55 (0.51, 0.60)*	0.57 (0.52–0.62)*
No ulcer	13,206	1798 (13.6)	–	–

^a^Adjusted for age, sex, duration of diabetes, treatment, HbA_1c_, history of cardiovascular disease, GFR measurement

*p < 0.05 vs no ulcer by *χ*^2^ test

**Table 3:** Unadjusted and adjusted estimates of the association between the covariates and amputation-free survival in people identified as having high-risk of foot ulceration

Variable	Unadjusted time ratio (95% CI)	Adjusted time ratio (95% CI)
Age at baseline	0.96 (0.96, 0.97)*	0.96 (0.96, 0.96)*
Sex		
Female	Ref.	Ref.
Male	1.06 (0.99, 1.13)	1.17 (1.09, 1.25)*
Duration of diabetes	1.00 (1.00, 1.00)*	1.00 (1.00, 1.00)*
Treatment		
Lifestyle	Ref.	Ref.
Insulin	0.82 (0.75, 0.89)*	0.79 (0.72, 0.86)*
Tablets/GLP-1 agonist	0.96 (0.96, 1.04)	0.89 (0.82, 0.97)*
HbA_1c_	1.00 (0.99, 1.02)	1.00 (0.99, 1.00)*
CVD		
No history of CVD	Ref.	Ref.
History of CVD	0.61 (0.57, 0.65)*	0.70 (0.65, 0.75)*
eGFR		
>60 ml min^−1^ [1.73 m]^−2^	Ref.	Ref.
30–60 ml min^−1^ [1.73 m]^−2^	0.81 (0.75, 0.87)*	0.95 (0.88, 1.02)
<30 ml min^−1^ [1.73 m]^−2^	0.42 (0.37, 0.48)*	0.56 (0.49, 0.63)*

*p < 0.05 by *χ*^2^ test

CVD, cardiovascular disease; Ref., reference

### Survival analysis: amputation as an independent event

The main adverse outcome in this study was death. We thus examined amputation as an independent outcome for people with diabetes who had high-risk of foot ulceration. People with diabetes who had an active ulcer at baseline had significantly higher risk of amputation compared with those who did not have an ulcer (HR 1.64 [95% CI 1.17, 2.28], *p* = 0.004) and with those who had healed ulcer at baseline (HR 2.24 [95% CI 1.37, 3.68], *p* = 0.001). There was no significant difference in risk between people who had a healed ulcer at baseline and people who did not have an ulcer (*p* = 0.138).

## Discussion

The aim of this study was to compare amputation-free survival rate in all people with diabetes at high risk of foot ulceration and not just all people with diabetes or just those with current ulcers or previous amputation. We also aimed to compare outcomes for different categories of high risk. The crude 2 year amputation-free survival rate in all people with high-risk foot was 84.5% and was 81.6% in the active ulcer group and 76.1% in the healed ulcer group. Thus, people with diabetes and previously healed ulcers had a worse outcome than people with an active ulcer, who in turn had a worse outcome than people with diabetes and no previous foot ulcer. One in four people with a previously healed ulcer died within 2 years, while the corresponding figure for people with no previous ulcer but with high-risk foot was one in eight.

Co-existing renal failure and cardiovascular disease were the main drivers for this poor outcome. When amputation was looked at as an independent endpoint, people with active ulcers were more than twice as likely to undergo amputation than those with previous ulcers. This may be because some clinicians readily choose digit amputation for toe ulcers with osteomyelitis. Interestingly, the risk of amputation for people with diabetes who had previous foot ulcers which had healed was the same as for people with high-risk foot who had never had an ulcer. However, this may be because individuals in the former group die before undergoing any amputation and may point to the importance of managing cardiovascular risk factors more aggressively for people with diabetes prior to and subsequent to developing foot ulcer [29]. Earlier studies have shown that previous ulcers are more likely to predict subsequent ulceration compared with other individual risk factors such as neuropathy or peripheral vascular disease [17–20, 22]. However, we have previously demonstrated that individual risk factors, even previous ulceration, are not as sensitive as a global risk score in predicting future foot ulcers [23].

A number of independent risk factors were identified as being associated with increased risk of death and amputation, all of which were unsurprising and included increased age, male sex, increased duration of diabetes, treatment with insulin, poor glycaemic control and history of cardiovascular and renal disease. Unfortunately, we did not have reliable data on smoking, diet or physical activity. Although healing rates for people with active ulcers have been reported to be 65–75% [18, 25–28], few studies have reported amputation-free survival. In 2014, Won et al [16] reported that the 1 year amputation-free survival rate in people with diabetes foot ulcers was 65.9% and that severity of ulcer and peripheral artery disease were risk factors for amputation. Winkley et al reported a similar rate of 69% at 18 months for individuals with ulcers [9], with older age, ischaemia, ulcer severity and poor glycaemic control all predicting adverse outcomes. Our amputation-free survival rate for individuals with high-risk foot, the majority of whom had no history of an ulcer, was higher at 84.5%, and our study was a population-based study including people whose ulcers were managed in the community as well as in hospital. To the best of our knowledge, our study is the first to investigate amputation-free survival rate and the risk associated in all people with diabetes who had high-risk foot (as opposed to just those with active ulceration) and is the first to compare different subcategories of high risk.

The risks of diabetes-related amputation have previously been shown to be associated with male sex, ethnic group and deprivation [30], resulting in 5 year mortality rates as high as 34% [31]. We have previously compared diabetes-related amputation with non-diabetes-related amputation [32]: the median times to death were 27 and 47 months, respectively, and cardiac failure explained the main difference in outcomes. Looking at these outcomes together with the results of the current study, it is likely that the poor outcomes in individuals with high-risk foot are related to social deprivation, sex, ethnicity, renal failure and cardiovascular disease (especially cardiac failure).

A weakness of this study is that it was an observational study using routinely collected clinical data and therefore causality cannot be attributed to any associations found. It is possible that some endpoints may have been missed but it is not anticipated that this would be greater in any particular individual subgroup. Data on smoking, a key risk factor for cardiovascular death and amputation, were not available. Furthermore, amputation rates were based on total amputations rather than major and minor amputations separately. We excluded people with prior amputations in order to look at incident events. This might explain why our ulceration rate, at 0.7%, was lower than the expected rate of around 1.7% [22]. If individuals with prior amputations had been included, it is likely that the majority of these people would have been categorised in the ‘previous ulcer’ group. Many might also have been in the ‘active ulcer’ group, since ulcers pre-date most amputations in the diabetic foot [1]. Hence, it is likely that the differences in outcomes between each of the three exposure groups would have been exaggerated if individuals with prior amputation were included. The data do, however, represent normal routine practice and was collected across Scotland from all 14 Health Boards, each of which will have had some variation in clinical practice. The results are thus likely to be relevant to clinical practice in developed countries.

The current study demonstrates that in people with diabetes who have high-risk foot, the risk of premature death was up to nine times the risk of amputation. Individuals with previous foot ulcers were at greater risk of death during follow-up than those with active ulcers. Those with diabetes with active ulcers had a higher risk of amputation than those with previously healed ulcers.

## Electronic supplementary material

ESM (PDF 137 kb)

## Data Availability

All data are held by Scottish Diabetes Research Network (SDRN). It is available on request from SDRN.

## Abbreviations

AFT: Accelerated failure time
AIC: Akaike’s information criterion
GLP-1: Glucagon-like peptide-1
NHS: National Health Service
SCI: Scottish Care Information
