# Supplementary material for: Amputation-free survival in 17,353 people at high risk for foot ulceration in diabetes: a national observational study
Source: Diabetologia. 2018 Aug 31;61(12):2590–7. doi: 10.1007/s00125-018-4723-y (PMC6223842; doi:10.1007/s00125-018-4723-y)
Supplement: Supplementary file 1 — (PDF 137 kb) [file 125_2018_4723_MOESM1_ESM.pdf]

ESM Figure 1: Plot to assess the proportional hazard assumption of ulcer groups using log negative-log survival curves.

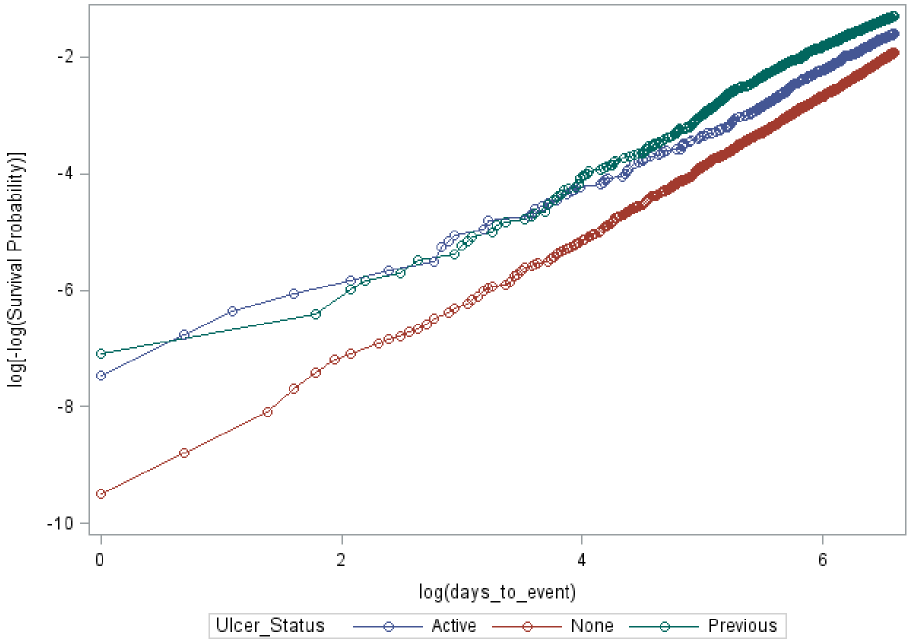

ESM Figure 2: Standardized Score Processes for active and healed ulcer group by Survival Time.

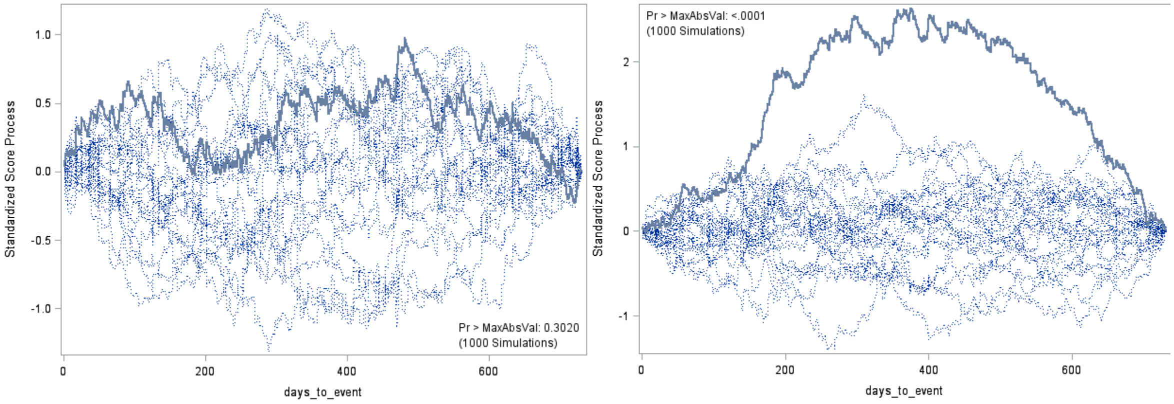

ESM Table 1: Analysis of Maximum Likelihood Estimates

**Parameter**		**Chi-Square**	**Pr > ChiSq**	**Hazard Ratio**
Ulcer status	Active	22.56	***<0.0001***	1.83
	Healed	134.93	***<0.0001***	3.07
Active ulcer*time		5.31	***0.0212***	1.00
Healed ulcer*time		30.48	***<0.0001***	1.00

ESM Table 2: AIC statistics for the all the distributions

Distribution	AIC
Gamma	18533.27
Log logistic	18528.14
Weibull	18533.78
Exponential	18610.76
Log normal	18668.69
